# HRM and the COVID‐19 pandemic: How can we stop making a bad situation worse?

**DOI:** 10.1111/1748-8583.12344

**Published:** 2021-03-11

**Authors:** Mark Butterick, Andy Charlwood

**Affiliations:** ^1^ Work and Employment Relations Division University of Leeds Leeds UK

**Keywords:** COVID‐19 pandemic, human resource management

## Abstract

This provocation argues that the COVID‐19 pandemic has exposed deep labour market inequalities. Partially underpinning these inequalities are human resource management (HRM) theories and practices which encourage and legitimise the commodification of labour. Workers whose jobs have been commodified have suffered disproportionately during the pandemic. While HRM is not wholly responsible for this suffering it is important for those of us involved in researching, teaching and practicing HRM to reflect on the ways in which what we do has made a bad situation worse so that we can do better in the future.

**Abbreviations**
CIPD: Chartered Institute of Personnel and Development
HRM: Human Resource Management

## 1 INTRODUCTION

In a few short months from January 2020, severe acute respiratory syndrome coronavirus 2, the virus that causes COVID‐19, transformed the world of work. Millions of workers lost their jobs or were placed on government support schemes as businesses and consumers changed their behaviour in response to the risks of contracting the virus. Millions more radically changed the ways in which they work, moving from offices to their homes for the same reasons. Further millions faced increased risks of contracting COVID‐19 as a result of their working conditions. The subsequent challenges for those involved in managing people have been huge and efforts to rise to these challenges have often been heroic.

**Practitioner notes**

**What is currently known**

- The COVID‐19 pandemic has convulsed the world of work, leading to a number of challenges for human resources (HRs).
- There have been significant inequalities in how different groups of workers have experienced the economic dislocation caused by the COVID‐19 pandemic.

**What this paper adds**

- The paper analyses the role of human resource management (HRM) theory and practice in bringing about the workplace inequalities that the pandemic has exposed.
- It argues that theories like ‘the flexible firm' model and the ‘HR architecture' model have played a role in creating and legitimising these inequalities.

**Implications for practitioners**

- HRM professional bodies espouse ethical codes and frameworks underpinning HR work, but such codes are widely ignored. HR professionals need to decide whether professional ethical codes mean anything and if they do, how can they be better enforced and upheld?

The efforts and heroism involved in people management during the COVID‐19 pandemic should not distract us from seeking to understand the role that human resource management (HRM) theory and practice has played in the unequal ways in which the pandemic's costs have been distributed within societies. It would clearly be hyperbolic to attribute responsibility for many of the pandemics negative economic and social consequences to people management practice. Poor judgement on the part of policy makers (Valizade et al., 2021), public health officials too slow to react to rapidly emerging scientific evidence, and the nature of the virus itself are all clearly more significant. Nevertheless, it is important for those of us involved in researching, teaching and practicing HRM to reflect on the ways in which what we do has contributed to making a bad situation worse so that we can do better in the future.

The article begins by arguing that some aspects of HRM theory (Atinkson, 1984; Lepak & Snell, 1999) have acted as transmission mechanisms for wider political and economic forces driving persistent economic inequalities and legitimising the increasing commodification of labour. Pervasive ideological individualism (Dundon & Rafferty, 2018) has contributed to widespread amorality (Quade et al., 2020) in people management. It then outlines how the contours of inequality that these forces have created have been revealed by the COVID‐19 pandemic before considering what can be done to create a better future for our profession and those it manages.

## 2 HRM THEORY, PRACTICE AND ECONOMIC INEQUALITY

Economic inequality in advanced industrial countries has increased dramatically since the early 1980s and is now at historically high levels (A. B. Atkinson, 2015). Underpinning this ‘inequality turn' are a range of broad economic and political forces; liberalisation of former state controlled economies leading to a doubling of the global workforce (Freeman, 2005); technological changes that have reduced the number of skilled but routine jobs (Autor et al., 2003); political changes that have weakened workers and union bargaining power (Damiani et al., 2018; Stansbury & Summers, 2020) and changes to the management and control of firms (Greenwald et al., 2019; Lazonick & O'Sullivan, 2000; Liu et al., 2014). HRM practices and the theory that underpins them have been one of several mechanisms through which these forces have acted (A. B. Atkinson, 2015; Bapuji et al., 2020; Bidwell et al., 2013; Dundon & Rafferty, 2018). HRM can both amplify and moderate increasing inequality (Bidwell et al., 2013; Cumming et al., 2020) but over the last 30 years, changes to HRM practice have tended to amplify rather than moderate inequality as market influences have become increasingly prominent in shaping HRM practice (Willman & Pepper, 2020). Two specific examples can illustrate how HRM theory and practice has contributed to rising workplace inequalities: The flexible firm model (J. Atkinson, 1984) and the human resource (HR) architecture model (Lepak & Snell, 1999).

The flexible firm model (J. Atkinson, 1984) showed how firms can differentiate between a secure core workforce and two levels of peripheral workers. The first with less job security, lower pay and limited access to career opportunities. The second typically low paid, part‐time and short‐term contract workers. Atkinson argued that this model allows firms to balance flexibility in production or service delivery with tight control of labour costs. Atkinson's ideas now underpin normative approaches to workforce resourcing. Particularly in firms that employ workers in jobs that require little skill or training (Bidwell et al., 2013; Forde & Slater, 2006; Koene et al., 2004).

The HR architecture model (Lepak & Snell, 1999) presents a normative theory of when firms should subcontract labour. The model segments workers into four quadrants on the basis of the value and uniqueness of workers' human capital. Where the uniqueness of human capital is low (i.e., where workers are doing jobs where skills to do the job can be developed quickly or where there is a plentiful supply of workers who can do the jobs) the model suggests that employment relationships should either be market‐based or contracted out. These employment relationships become transactional with little training, job security and lower rates of pay. By contrast, where knowledge is both unique to the firm and valuable the model advocates secure, well‐rewarded long‐term employment (if knowledge is unique but not strategically valuable, work can be subcontracted to professional service firms). The logical consequence of the model is that firms who had previously made use of encompassing internal labour markets which provided a degree of security and career structure for all workers would do better if they commodify part of their workforce. The impact of subcontracting is evidenced by the finding that one‐third of workers working in US corporations are not employees of the company they are working in (Cappelli, 2020).

These models and associated people management practices are promoted through HR education and HR professional bodies. For example, the UK's Chartered Institute for Personnel and Development, has ‘flexibility' as part of the core curriculum that aspiring members need to study and demonstrate knowledge of to qualify for membership. While the precise nature of what constitutes flexibility is not specified in the Chartered Institute of Personnel and Development's (CIPD) curriculum outline and Atkinson's model is not the only approach to achieving flexibility that is taught, in our experience CIPD textbooks and approved courses taught by colleges and University business schools typically include content on Atkinson's model which is often presented normatively with minimal critical consideration of the consequences for workers. HR professional bodies in other Anglophone nations promote these ideas less directly, but all place the need for ‘business acumen' (SHRM, 2014) or to be ‘business driven' (AHRI, 2016) at the centre of their professional standards and competencies. This bottom‐line driven logic is inexorably driving processes of labour commodification (Davenport, 2018).

The broad point here is that HR acts as a servant and propagator of labour commodification (B. Kaufman, 2015). Resulting in ways of organising employment that might be considered optimal from a shareholder perspective but which are unlikely to be optimal for the workers exposed to flexibility and subcontracted market‐based employment relationships. Such people management practices existed before the development of the theories discussed above and would doubtless exist without them. Nevertheless, the theories provide ideological support to management practices that promote inequality, legitimating the idea that it is necessary to commodify labour for firms to be considered ‘efficient' (Spencer, 2020).

While the broad economic and managerial forces described above underpin the drive towards labour commodification, the managers who have implemented decisions that commodify labour are not automatons driven by these forces, they retain choices about how they respond to them (Archer, 2007; Sayer, 2011). Why have they behaved in this way? Dundon and Rafferty (2018) frame the changes as being driven by ‘ideological individualism' and trace this to a particular reading of the works of Adam Smith, which they associate with Friedman (1970). This ethical framing results in managers downplaying or ignoring ethical considerations. Arguably, the result of this is not so much consciously immoral decisions and actions on the part of managers, but amorality, where ethics are not considered in day to day management activities (Quade et al., 2020). The next sections will argue that the consequences of this behaviour have been starkly revealed during the COVID‐19 pandemic.

## 3 WORK, INEQUALITY AND THE EXPERIENCE OF THE PANDEMIC

At the outset of the pandemic the World Health Organization advised national governments to control the spread of COVID‐19 through widespread testing, contact tracing to find and test those who had come into contact with the virus combined with systems to isolate the infected so that they could not spread the virus further. The speed with which COVID‐19 spread took governments by surprise with the result that most were unable to mobilise the capacity to immediately follow this advice. Consequently, many countries issued ‘stay‐at‐home' orders banning all but essential social and economic activity outside of the household. These orders had an immediate impact on work; unemployment increased; home working increased; some workers were placed on short‐time working schemes or were furloughed with government support. There were significant inequalities in who experienced job loss and loss of income compared to continuing to work from home while being paid as normal.

Patterns shaped by the application of the ideas about workforce strategy encapsulated by the flexible firm and HR architecture models are clearly visible in these inequalities, particularly in the United Kingdom and United States of America. By late April 2020 20% of workers in the United States of America and 17% of workers in the United Kingdom had been laid off. One key indicator of lay‐off risk is doing a job where fewer than 40% of tasks could be completed at home (Adams‐Prassl et al., 2020). Almost all of those doing jobs involving tasks that could be completed from home were in occupations that would, following the prescriptions of the HR architecture model, likely benefit from a ‘commitment‐based' approach to HR: managers, technical workers and professionals in IT, finance and operations, design, architecture, engineering and the law. Office administrators and support workers were the only nonessential occupational group likely to appear in one of the more market‐based forms of employment relationship advocated by the model for whom working from home was common.

Workers who could not work from home have typically been treated as disposable commodities as have those on temporary contracts. Workers who were normally expected to be flexible in their hours of work were also more likely to experience loss of income (Adams‐Prassl et al., 2020). While this general pattern has appeared in many countries, there were significant and interesting variations between countries (Valizade et al., 2021). For example in Germany, long standing arrangements to reduce working time as an alternative to unemployment reduced the extent of lay‐offs and ensured that a much lower proportion of workers who were unable to work from home lost their jobs (Adams‐Prassl et al., 2020). More broadly, countries with greater coverage of collective bargaining prior to the pandemic provided more income support to workers affected by the pandemic (Valizade et al., 2021). Differences such as these vividly illustrate the importance of national institutions and systems of labour market regulation in moderating trends towards the commodification of labour and workers' exposure to flexible working practices that led to increased risk of job loss and income insecurity during the pandemic.

## 4 HRM INEQUALITIES AND HEALTH RISKS

Another revealing feature of the pandemic labour market is that lower paid workers in commodified jobs also featured disproportionately in lists of ‘essential' workers who continued to work as normal through periods where much other economic activity was shut down through stay‐at‐home orders. Low paid ‘essential' worker occupations included those working in food production, distribution and sales, care workers and ancillary staff in the health and social care sectors as well as workers involved in security and transport more generally. This raises questions about the social legitimacy of the HRM practices that produced such large divergences between these workers' (low) market value and (high) value to society (Winton & Howcroft, 2020). Societies will have to reckon with these questions in the postpandemic world. There are also more immediate questions of social justice. Workers in many ‘essential' occupations were also significantly more likely to get sick and to die as a result of COVID‐19 than the rest of the working age population.

Food processing factories have proved to be particular hotspots for virus outbreaks. Environmental conditions in these factories make it especially difficult to prevent COVID‐19 transmission if a worker is infectious, because the virus is able to survive for longer in cooled air and cooled air carrying the virus is recirculated by energy efficient ventilation systems. This means that large‐scale outbreaks are likely even if the employer behaves responsibly by working closely with public health authorities to test workers while providing sick pay and job security for workers who are ill or who have been exposed to the virus (Guenther et al., 2020). However, it is also clear that many employers have not even tried to behave responsibly and that HR policies of not providing sick pay, expecting workers to turn up for work when sick under the threat of dismissal and use of temporary staff made the pandemic worsen (e.g., Bland & Kelly, 2020; Laughland & Holpuch, 2020; ONS, 2020b). In the USA, the United Food and Commercial Workers International Union estimated that at least 225 workers in food production, distribution and retail jobs had died as a result of COVID‐19 as of early July (Samaha et al., 2020).

Evidence from the United Kingdom (ONS, 2020a) allows us to quantify the increased risks faced by essential workers and those who could not work from home. Risk of death from COVID‐19 was higher for men in 17 occupations including care work; cleaning machinery and packing goods in factories; security guards; bus, van and taxi drivers; chefs; sales and retail assistants; food manufacturing; vehicle technicians, mechanics and electricians as it was for women in four occupational groups, care workers; retails and sales assistants; clerical workers in the civil service. On the basis of the available evidence it is not possible to say authoritatively why workers in these occupations were more likely to die from COVID‐19, but a plausible explanation would be that working environments increased risk of exposure while lower pay and job insecurity may also have contributed to living conditions (shared and multi‐generational households) that make transmission more likely. The nature of work and employment relationships (low pay, low job autonomy and job insecurity) also contribute to poorer general health (Chandola & Zhang, 2018; Wilkinson & Marmot, 2003) which could have made the severity of infection worse. Although this evidence is circumstantial, it nevertheless suggests that the application of HR policies and practices which commodify low skill work has resulted in a deeply unfair distribution of the human suffering arising from the pandemic.

This has been a tragedy for those who have died or suffered bereavement but the spread of COVID‐19 through workplace outbreaks has had wider costs for society, making it harder for public health authorities to contain and control the spread of the virus. Examples of this phenomena abound. Use of agency staff and lack of sick pay contributed to the spread of COVID‐19 in care homes in the United Kingdom (ONS, 2020b). In Agriculture, food processing and distribution in the United States of America (Ho, 2020; Laughland & Holpuch, 2020; Samaha et al., 2020), textiles and clothing factories in the United Kingdom and United States of America (Bland & Kelly, 2020; Miller, 2020), and warehouses in South Korea (Lee & Jin, 2020). In Singapore, a second wave of infection spread as a result of poor working conditions and unsanitary cramped dormitories used to house low paid migrant construction and service workers (Ratcliffe, 2020).

Many of the examples above are from sectors where people management is characterised by informality so labour commodification is unlikely to have been informed by HRM theories taught on business school curriculums (although the growth of these sorts of employers has been partly driven by subcontracting arrangements replacing in‐house manufacturing in the food and clothing sectors). This reinforces the argument that HRM theory is not directly responsible for labour commodification and its consequences during the pandemic (Spencer, 2020). Nevertheless, the evidence above is also replete with examples of ‘amoral management' (Quade et al., 2020). Managers have not been consciously immoral when taking actions that have exposed workers and societies to increased health risks, rather these have been decisions taken without adequate consideration of their ethical consequences. How can HR stop legitimating and implementing labour commodification and challenge the ubiquity of amoral management behaviour?

## 5 WHAT NEXT FOR HR?

How does the field of HRM escape the increasingly dystopian people management landscape that the pandemic has revealed? In answering this question, it is first necessary to make an obvious but nevertheless important point. An article like this does not have and cannot hope to have all the answers. Above all we need to talk to each other about the challenges ahead, to share ideas and experiences that will allow us to collectively attempt to define and bring about a better future for our field. These conversations need to happen in academic associations, professional bodies and with those who belong to neither; workers, unions, managers from outside the HR field, policy makers and government. With this caveat in mind, what actions can we take, as academics and practitioners, to row back from the problems with people management that the pandemic has exposed?

Peter Cheese, chief executive of the CIPD has also posited that improvements will happen organically because the pandemic has led organisations to focus on their duties of care towards their workers and worker wellbeing to a significantly greater extent (Warren, 2020). This view is unrealistic. It is not as if the public were unaware of some of the bad employment practices that the pandemic has highlighted, but public opprobrium on its own is rarely enough to force employers to change en masse. At best bad publicity might bring about changes in the organisations that are its focus while more anonymous subcontractors continue as before. Further, rising unemployment as a result of the pandemic will hand employers more labour market power, creating more opportunities for the commodification of labour. Therefore, we cannot expect public opinion and changing employer sentiment to act as deus ex machina that will right the injustices of modern employment practices and reverse trends towards the commodification of labour. What are the alternatives?

Following from our analysis above, it is important first to note the deep‐rooted structural forces driving the commodification of labour (Dundon & Rafferty, 2018; Spencer, 2020; Thompson, 2011). However, structural forces can be challenged and changed. Differences in COVIDs labour market consequences between countries discussed above point to the importance of national institutions in moderating the extent of labour commodification. It is therefore important to make the case for policy and regulation that decommodifies labour and improves job quality because such regulations creates space for HRM practices which provide job security, good work and worker voice (Forde et al., 2020; Warhurst & Knox, 2020). There are formidable challenges involved in bringing about such structural change (Spencer, 2020) but ultimately, some campaigns to improve job quality can succeed because we are not automatons driven purely by forces we cannot control but moral and ethical beings with a degree of agency over how we respond to our environment (Archer, 2007; Sayer, 2011).

Sayer (2011, pp. 145–146) argues that the claim that we are ethical beings is partly based on empirical observation of how people behave and partly a hope. He argues that lay ethical practice is always flawed because there is no universal ethical code that will always result in ethical behaviour and outcomes. The possibility of ethical behaviour depends on the possibility of unethical behaviour. Rather than looking for a perfect set of abstract ethical principles, we should look to expand the degree to which we behave ethically by focussing on the avoidance of harm and promotion of human flourishing as outcomes. Sayer's ideas parallel some of Karen Legge's arguments about how HRM can be practiced ethically. It is not always possible to manage employees in ways that always avoid harm and promote flourishing, but it is important for managers to confront the moral implications of their actions (Legge, 1998, p. 169). In doing so they will experience emotions (moral sentiments) that will incline them to behave ethically as far as possible (Sayer, 2011, p. 146). In short, those of us involved in managing people must display a commitment to promoting flourishing and avoiding harm and to do this, we must be constantly aware of the ethical dimensions to our management practice.

This is something that those of us who teach HRM can contribute to by ensuring that ethics is a central part of the courses we teach. Writing this article has prompted one of the authors to reflect that while ethics has always been covered in their teaching. It was taught as a discrete area, often at the end of the course, rather than as something that touches on all areas of HR activity. They have revised their approach to teaching as a result.

Ethical practice can also be promoted through professional norms and rules. HR professional bodies in the United States of America (SHRM, 2014), United Kingdom (CIPD, 2020), Australia (AHRI, 2016) and Canada (CPHR, 2016) have professional codes that include definitions of what it means to be ethical. These codes specify that HR professionals should act in a way that promotes or advances principles of human dignity and justice. However, there is little evidence that these ethical codes are enforced in any way. Many of the management practices implemented by members of these professional bodies clearly undermine human dignity and result in injustices. What is the point of professional standards if there are no enforcement mechanisms to ensure that HR practitioners adhere to them? Ethical codes need to be backed with action in the form of public complaints processes, exclusion from membership for those who transgress ethical standards and boycotts of companies that systematically violate professional ethical standards.

Ethical conduct is not simply a matter for individuals. Organisations undertake institutional work that seeks to maintain, shape or disrupt the institutions that regulate the employment relationship (Bapuji et al., 2020). Professional bodies can be a louder voice in public debates for regulation and institutions that improves job quality and so enhance human dignity. They can also more overtly challenge businesses and organisations that undertake institutional work that aims to have the opposite effect by promoting practices that commodify labour.

Professional bodies can also reconsider the content of the curriculums that aspirant members are required to study. Does a necessary focus on ‘business acumen' and topics such as strategic HRM and performance drive out or come at the expense of more pluralistic models of HR that emphasise the importance of how HR impacts on stakeholders? This is important because different paradigm models of HRM lead to quite different conclusions about the state of HRM and its consequences (Kaufman et al., 2021). It is therefore important for professional bodies to promote models that recognise the importance and legitimacy of stakeholder interests.

The question this article poses is ‘how can we stop making a bad situation worse?' The question applies to our academic research too. In answering this question it is important to recognise that some strands of management and HRM research provide legitimation to practices that commodify labour and increase inequality. They do this by focussing on managerial concerns of efficiency, productivity and financial performance (none of which are necessarily bad or undesirable) *without considering the distributional and welfare consequences* of the practices and outcomes which they study (Kaufman, 2020). The result is amoral management research which mirrors the amoral management decisions and behaviour it often studies.

A number of recent scholarly contributions have recognised and discussed these issues (Bapuji et al., 2020; Bidwell et al., 2013; Dundon & Rafferty, 2018; Forde et al., 2020; Godard, 2014; Kaufman, 2015, 2020; Kniffin et al., 2021; Vincent et al., 2020). Given the brevity of the provocations format we will not repeat all of the suggestions these articles make for future HRM research that is focused on understanding the forces that shape workplace inequalities; but we will briefly amplify ideas and suggestions that seem to us to be particularly pertinent for the postpandemic world.

It is important to research HR professionals as actors in the system of HRM (Bidwell et al., 2013). If research is to contribute to halting the reproductive cycle of labour commodification, we need research that shows and explains how HR and other people managers make strategies and decisions about how they manage their workforces. What leads them to pursue approaches that result in commodification? Why do organisations that do not follow a commodification approach do things differently?

It is important to study the distributional and welfare consequences of evolving approaches to managing people and organising employment and to study these questions at multiple levels of enquiry: individuals, organisations, industries and societies (Bapuji et al., 2020; Bidwell et al., 2013, 2020, p. 2020; Kaufman, 2020; Vincent et al., 2020). The pandemic has shone a light on three particular areas which seem to us a priority for further research if academics are to shed light on what can be done to address the workplace and labour market inequities the pandemic has highlighted. First, the role and status of migrant workers, important given the evidence discussed above that migrant workers have often borne the brunt of the pandemic. What can be done to reduce their vulnerability to poor quality work?

Increasing use of technology to monitor and control worker behaviour. Evidence suggests that some employers have responded to widespread working from home with new digital tools for monitoring worker behaviour. One of the highest profile beneficiaries of the pandemic, Amazon, are well known for using such tools to monitor and enforce worker effort (Bloodworth, 2018; Bort, 2019). Will the pandemic result in such tools becoming more widely used, and if they are, what will the consequences be?

What are the longer term consequences of the rapid shift to home‐based working that the pandemic has presaged (Kniffin et al., 2021)? For many, the flexibility afforded by home‐working has been a liberation but there is already evidence that some employers see the continuance of home‐working as an opportunity to reduce labour costs (Comboye, 2020) and that women and minorities may be particularly disadvantaged by this shift (Topping, 2020).

The pandemic has exposed deep‐labour market and workplace inequalities linked to the widespread commodification of Labour. The policies that have caused these inequities were often implemented by HR professionals and in some cases followed theories and ideas developed and taught by HR academics. While there are clearly broad structural forces driving these changes that are difficult to challenge, all of us working in the field of HR whether as academics or practitioners face a choice. Do we want a future that continues and accelerates the race to the bottom HRM practices that the pandemic has so starkly revealed or will we work to bring about more fundamental changes to how businesses value and treat people?

## Data Availability

No data beyond the references were used in authoring this article.
